# Analysis of Weld Lines in Micro-Injection Molding

**DOI:** 10.3390/ma16176053

**Published:** 2023-09-03

**Authors:** Sara Liparoti, Giorgia De Piano, Rita Salomone, Roberto Pantani

**Affiliations:** Department of Industrial Engineering, University of Salerno, via Giovanni Paolo II 132, 84084 Fisciano, Italy; gdepiano@unisa.it (G.D.P.); rsalomone@unisa.it (R.S.); rpantani@unisa.it (R.P.)

**Keywords:** micro-injection molding, weld lines, tensile modulus, Moldflow simulations

## Abstract

Micro-injection molding (µIM) is a widespread process for the production of plastic parts with at least one dimension, or feature, in the microscale (conventionally below 500 µm). Despite injection molding being recognized as a robust process for obtaining parts with high geometry accuracy, one last occurrence remains a challenge in micro-injection molding, especially when junctions are present on the parts: the so-called weld lines. As weld lines are crucial in determining mechanical part performances, it is mandatory to clarify weld line position and characteristics, especially at the industrial scale during mold design, to limit failure causes. Many works deal with weld lines and their dependence on processing parameters for conventional injection molding, but only a few works focus on the weld line in µIM. This work examines the influence of mold temperature on the weld line position and strength by both experimental and simulation approaches in µIM. At mold temperatures below 100 °C, only short shots were obtained in the chosen cavity. At increased mold temperatures, weld lines show up to a 40% decrease in the whole length, and the overall tensile modulus doubles. This finding can be attributed to the reduction of the orientation at the weld line location favored by high mold temperatures. Moldflow simulations consistently reproduce the main features of the process, weld line position and length. The discrepancy between experimental and simulated results was attributed to the fact that crystallization in flow conditions was not accounted for in the model.

## 1. Introduction

Nowadays, miniaturized products have gained increasing interest in many industrial applications, involving microelectronics, automotive industries, telecommunication systems and medical engineering [1]. Micro-injection molding (µIM) is a manufacturing technology which enables the one-shot large-scale production of fine and precise parts having at least one dimension or feature in the micro/nanoscale [2]. The accuracy and the quality required by this process demand a complete understanding of typical molding problems, such as weakness of the weld line, voids, burning and nonuniform shrinkage, which are more evident in micro-injection molding parts than in conventional injection molding [3].

During the production process, the collision of two or more molten flow fronts and the subsequent solidification generates a region called the weld line, showing worse performances than the bulk material [4]. Molecular orientation induced by fountain flow and poor intermolecular entanglements across flow fronts are some of the factors responsible for the lower mechanical and optical properties of welded parts [5,6]. Crack initiation may occur at the weld line area due to a notch which can be formed by a high orientation if the two melt streams suddenly freeze [5,7,8].

The most common approaches used to reduce weld lines are the optimization of injection molding parameters, the enhancement of heating/cooling systems and the exploitation of special molding tool devices [7,9].

Xie et al. [8] designed and fabricated a special mold equipped with a system to enable fast mold heating/cooling. The variotherm system, consisting of electrical heaters and water-cooling channels, was adopted for obtaining micro-injection molded polypropylene parts; it was found that mold temperature is the most important factor affecting the filling process: the higher the mold temperature, the stronger the junction is. The mold took 120 °C to allow a complete filling for the adopted material. However, high mold temperatures often determine flash problems. Mold temperature strongly affects the parts’ performances; for instance, a high mold temperature (up to 150 °C for a polyamide) leads to higher tensile strength (it increases from 80 MPa to 95 MPa), while impact strength decreases (it decreases from 33 to 20 kJ/m^2^) [10]. Liao et al. [6] studied the microstructure and molecular orientation distribution at weld lines for a µIM linear low-density polyethylene. Mold temperature markedly affects the performance of parts. Also, the increase in injection speed and packing pressure may determine an increase in the tensile strength of welded products [8]. Another method for reinforcing weld lines is the introduction of an ultrasonic oscillator: when the ultrasonic energy can vibrate, the polymer melts without additional heating [11]. The tensile strength of injection-molded Nylon 6 nanocomposites increased by about 2% with a mold temperature increase from 60 °C to 100 °C, for all the considered cavity thicknesses [12]. Xie et al. [9] showed that the mechanical performances at weld lines increased when the ultrasonic energy was increased: the tensile stresses at the weld lines obtained with ultrasonic-assisted injection molding were 8.32–24.1% higher than the ones obtained with the conventional process. Material melt flow rate is another important aspect to consider when weld line effects are investigated. Purgleitner et al. [4] demonstrated that for conventional injection molding, the tensile modulus increases by about 10% with a 20 °C mold temperature increase. However, the influence of melt flow rate seems to be less significant than that of mold temperature. A possible explanation could be related to better flowability due to the lower viscosity, which induces higher entanglement numbers across the weld line area. 

The formation of weld lines and their location along the flow path is a well-studied phenomenon in conventional injection molding; however, only a few papers are devoted to micro-injection molding [11,13,14,15,16]. As the weld line can significantly lower the mechanical strength of µIM parts, it is relevant to clarify the position of weld lines and also to provide possible routes for limiting weld line formation. In this work, a variotherm system composed of heating rods and cooling channels was adopted for modulating mold temperatures during the micro-injection molding experiments. An isotactic polypropylene (iPP) was selected to conduct µIM tests at several mold temperatures with the aim of investigating the effect of this process parameter on the weld line formation and part mechanical properties. A simulation of µIM was also conducted to investigate the flow behavior during the process. 

## 2. Materials and Methods

### 2.1. Micro-Injection Molding Machine

**Figure 1:**
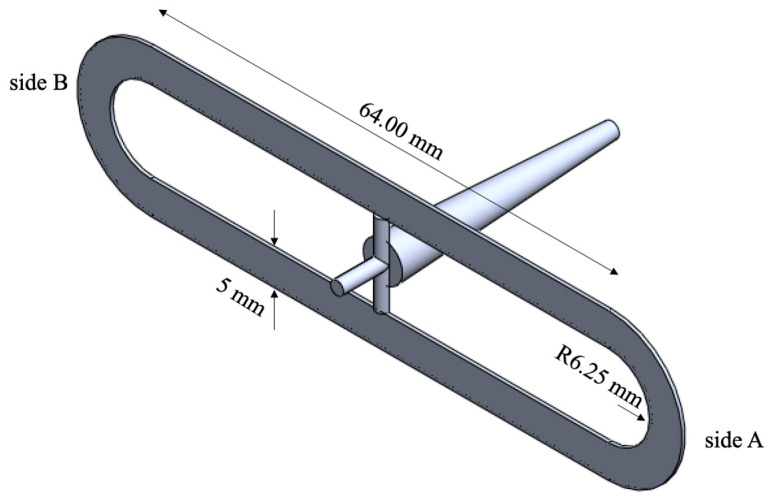
Sketch of the cavity adopted for the injection molding experiments.

Micro-injection molding machine (MegaTech H10/18-2, Tecnica Duebi, Fabriano, Ancona, Italy) was used to obtain specimens of iPP (T30G, Basell, Ferrara, Italy). The cavity adopted for the experiments is sketched below, cavity thickness is 0.5 mm (see Figure 1):

The following µIM parameters were used: 80 MPa was adopted as maximum injection pressure, 8.7 cm^3^/s as flow rate, 210 °C as melt temperature and several mold temperatures were set to study the effect of this parameter on the weld line properties. 

### 2.2. Specimen Characterization

The µIM specimens were observed with an optical microscope (Leica MZ6, Milan, Italy) in crossed polarized light to analyze the filling length and the weld line.

Mechanical properties in correspondence with the weld line and the continuous part were evaluated using stress–strain analyses conducted in a dynamic mechanical analyzer (PerkinElmer DMA8000, Buckinghamshire, UK). The dimensions of the specimens for stress–strain analyses were: 8 mm length, 4.5 mm width, and 0.5 mm thickness. The thickness value changed when analyses were conducted at the weld line location: in that position, the thickness was 0.45 mm. The tensile strength measurements were performed at 30 °C [17]. The set maximum load was 8 N, with a load rate of 1 N/min. Engineering stress, σ, and strain, ε, were calculated from Equations (1) and (2) [6]:

(1) $$\sigma =\frac{F}{A}\;$$

(2) $$\varepsilon =\frac{∆L}{L_{0}}$$

where F is the experimental static force (N), A is the initial cross-section (mm^2^), ∆L is the experimental static displacement (mm) and $L_{0}$ is the initial length between clamps (3 mm).

### 2.3. µIM Simulations

**Figure 2:**
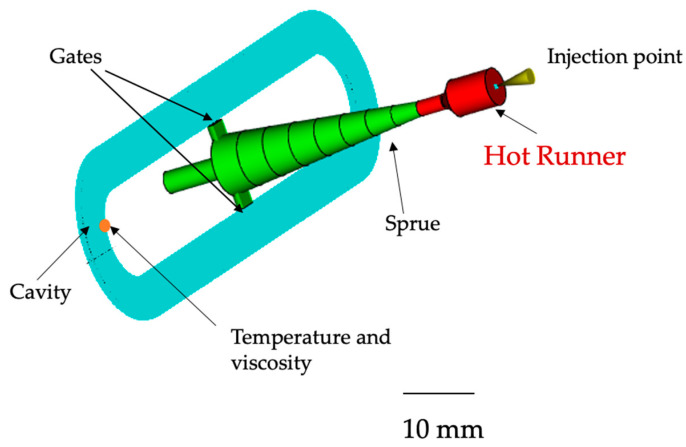
Cavity geometry implemented in MF simulations.

The µIM process was simulated by Autodesk Moldflow Insight 2021 (Autodesk, Inc., San Rafael, CA, USA), MF. A dual domain model (42,786 elements) was built and then meshed with triangle elements, having a global edge length of 2 mm and at least 10 elements over the thickness. The runner and the sprue were modeled as cold runner beam elements. The hot runner was also included to obtain more accurate simulation results. The mold block made of tool steel “P-20” was also included. A heat transfer coefficient of 25,000 W(m^2^K)^−1^ was adopted at the wall. Material database of T30G polypropylene (iPP) (adopted in this work) was created according to its characterization reported elsewhere [18]. Figure 2 shows the cavity geometry implemented in MF.

## 3. Results and Discussion

Mold temperature may affect the filling capacity of the melt, the residual orientation and crystallization. In their turn, these parameters affect mechanical performances, especially where weld lines form. Weld line formation in the injection molding process occurs when two or more melt flow fronts contact each other immediately after the cooling process. Thus, it is important to understand (i) if the melt is able to fill the cavity up to the weld line location, (ii) where the weld line forms, and (iii) if the weld line is a cold or flowing one. To this aim, the impact of mold temperature was evaluated concerning both the fill capacity and the strength of the weld line. These qualities were analyzed using optical microscopy and tensile tests. The influence of mold temperature on weld line location and weld line type was also assessed using simulations conducted with MF.

**Figure 3:**
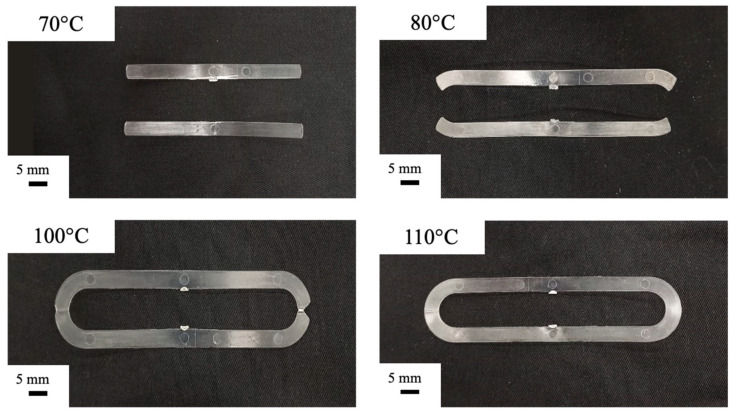
IM specimens obtained with several mold temperatures.

Figure 3 shows micrographs of the µIM specimens obtained with several mold temperatures. These pictures were adopted to evaluate the percentage of filled volume for each condition.

**Figure 4:**
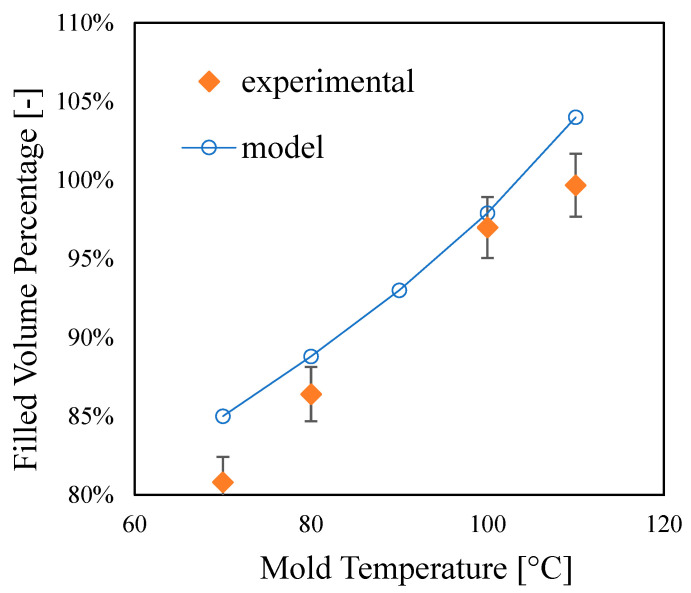
Experimentally evaluated data and model (Equation (3)) calculations of filling volume percentage at different mold temperatures.

As expected, the cavity volume filled by the polymer increases with the mold temperature increase, confirming that the mold temperature strongly affects the filling ability in the µIM process. The filling of the cavity represents a challenge for µIM due to the high aspect ratio [19]. Once the flow rate is sufficiently high to limit sudden solidification as much as possible [20], the mold temperature must be properly set. In the cases proposed in this work, a temperature higher than 100 °C is required to completely fill the cavity. Figure 4 shows the filling length evaluated at several mold temperatures.

The flow length increases with mold temperature. This result is consistent with those reported in the literature about flow filling length in micro-injection molding. It was reported that, with a settled 40 MPa filling pressure (that is lower than the pressure adopted in this work), a 140 °C mold temperature is necessary to obtain about a 40 mm filling length; furthermore, filling length improves with the increase in filling pressure [21]. 

In a previous work [21], a simple equation was provided to predict the final length, $L_{f}$, of a polymer flowing in a rectangular cavity of thickness H:

(3) $$L_{f}=\left(\frac{H}{2}\right){\left(\frac{H^{2}}{\alpha \left(m+2\right)}{\left(\frac{P}{K}\right)}^{m}{\left(1-\frac{2\delta }{H}\right)}^{m+2}\left(\frac{\delta }{2H}\right)\Theta \left(\frac{1}{\beta }\right)\right)}^{1/\left(m+1\right)}$$

in which *P* is the injection pressure, α the thermal diffusivity, δ is the solidified layer at the wall when the flow stops, and Θ is a dimensionless temperature equal to:

(4) $$\Theta =\frac{T_{inj}-T_{f}}{T_{f}-T_{w}}$$

where $T_{inj}$ is the injection temperature, $T_{f}$ the no-flow temperature and $T_{w}$ the mold temperature.

In Equation (3), the material is assumed to follow a power-law rheological equation, where the shear stress is given by Equation (5).

(5) $$\tau_{xy}=K{\left(-\frac{\partial v_{x}}{\partial y}\right)}^{1/m}$$

where $v_{x}$ is the velocity along the flow direction, x, and y is the thickness direction (0 = midplane, H/2 = wall).

In that work, for the same iPP adopted in this work, the no-flow temperature was set to 147 °C, $\frac{\delta }{H}=0.6$2 and β=1. The thermal diffusivity is $\alpha =7.5\times 10^{-8}\;m^{2}/s$. As for the rheological parameters, K was calculated at *P*/2 and $(T_{inj}+T_{m})/2$. Adopting the same approach, with the rheological parameters reported in the literature [18] and applying Equation (3) to our molding conditions in which it was assumed that the pressure was constant and equal to its maximum value, the flow-lengths predicted are reported in Table 1. Since the maximum flow length corresponding to a complete filling is 45.8 mm, the equation predicts the temperature needed for a complete filling well.

The same paper also provides an equation for predicting the solidification time, as reported in Equation (6):

(6) $$t_{f}=\frac{H^{2}}{\alpha }\Theta \frac{2\delta }{H}{\left(1-\frac{2\delta }{H}\right)}^{m+2}\frac{\left(m+2\right)\left(m+3\right)}{8\left(m+1\right)}$$

The values of solidification times predicted by Equation (6) are also reported in Table 1 and show that the times are very short (lower than 0.25 s). 

**Figure 5:**
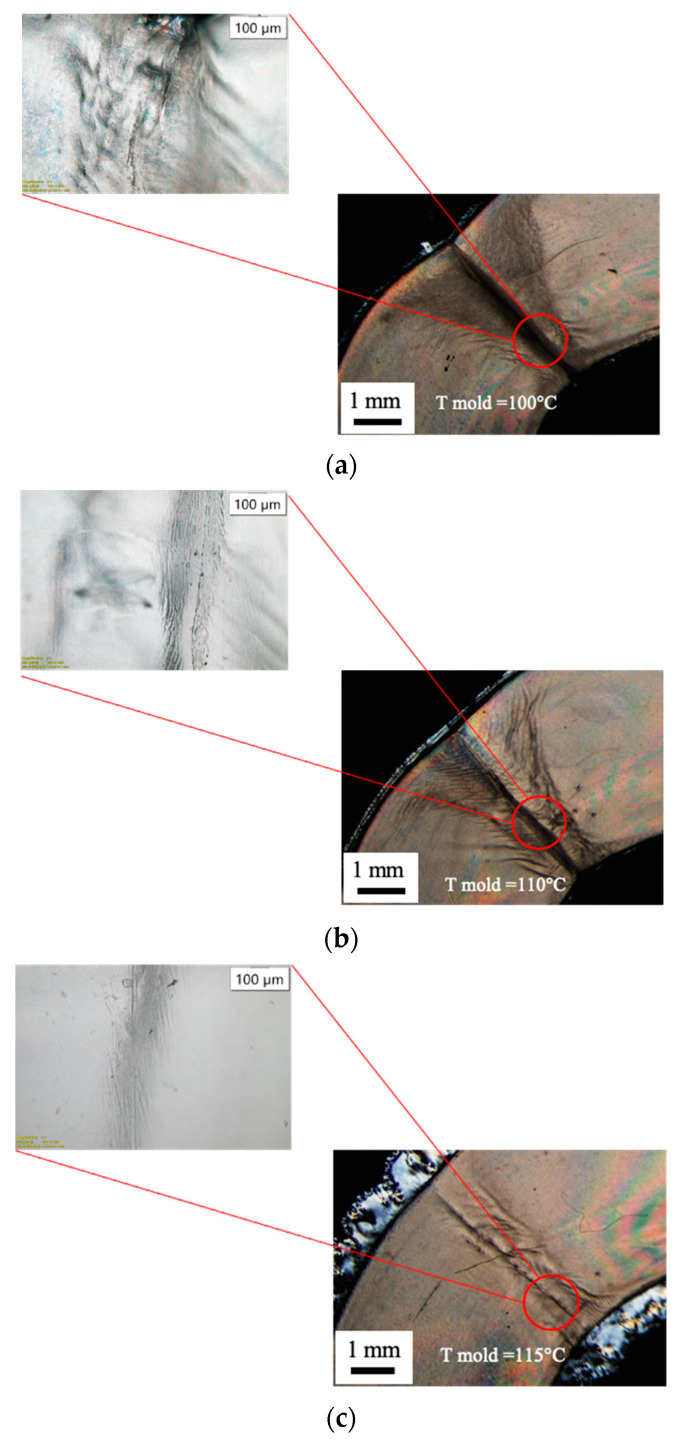
Optical micrographs of the µIM specimens obtained with 100 °C (**a**), 110 °C (**b**) and 115 °C (**c**) mold temperatures in the weld line area.

**Figure 6:**
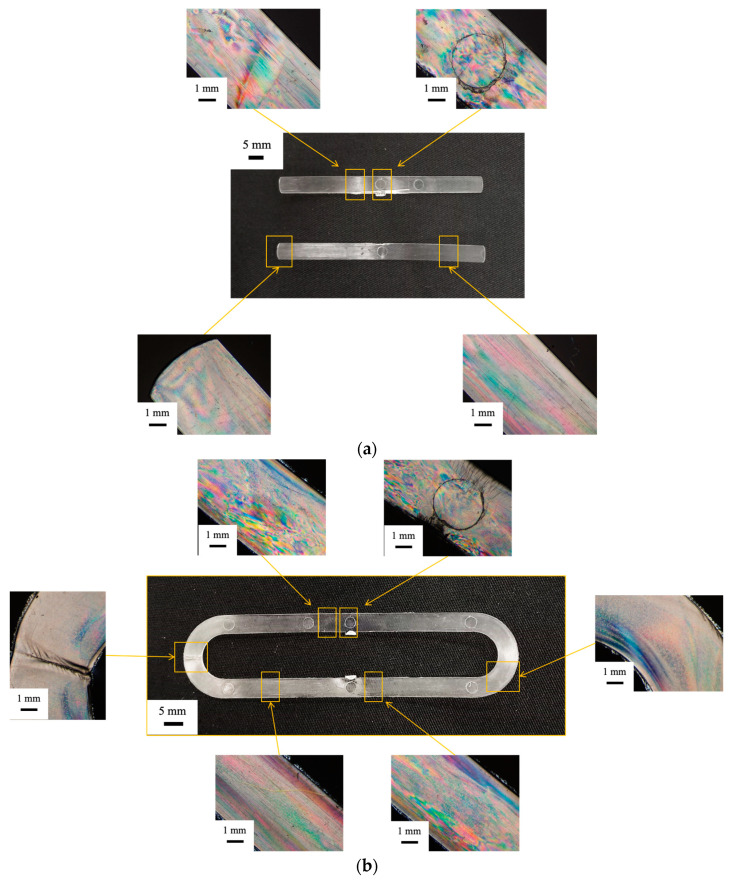
Optical micrographs in various parts of the µIM specimen obtained with mold temperatures of (**a**) 70 °C and (**b**) 110 °C.

Cavity filling must account for two additional phenomena with respect to cooling: the crystallization occurring already during the filling process and wall slip. Referring to the half-crystallization time of the adopted iPP, at least 20 s are required to achieve 50% crystallization in quiescent conditions [18]. During flow, the iPP crystallizes much faster [18] and this can anticipate solidification when low mold temperatures are adopted. Obviously, the fact that the cavity appears filled does not imply the formation of a strong weld line. Figure 3 also shows that it is not possible to select very high temperatures: the specimen obtained with a 115 °C mold temperature shows flashing due to the mold opening induced by high pressures inside the cavity and the low viscosity of the material which can thus enter the space between the two mold halves. Concerning wall slip, it is generally neglected in conventional injection molding, although it could play a significant role at the microscale [16]. Wall slip can be ascribed to the disentanglement of the bulk chains when attached to the mold walls and may increase filling length [15]. However, in the cases reported in this work, the wall slip phenomenon did not occur, as can be observed from the optical micrographs (Figure 5 and Figure 6): no sharkskin [22] is visible in any of the obtained specimens.

Due to the high-velocity gradient, molecules in the skin region experience an orientation in the flow direction. In the core, the flow is weak and molecules are stretched by the fountain flow and are then pushed towards the edge regions, ending up being parallel to the weld line [23]. In the literature, weld lines can be distinguished into two main types: cold or stagnating weld lines, formed by a head-on impingement of two melt fronts, and hot or flowing weld lines, occurring when two melt streams continue to flow after their lateral collision [4].

Figure 5 shows the optical micrographs of the parts obtained with mold temperatures high enough to allow weld line formation. A marked junction can be observed for the part obtained at 100 °C; it can be attributed to the presence of a cold weld line. Junctions become less marked at increased mold temperatures; such behavior is due to the formation of a flowing weld line, at least in certain sections of the part.

Figure 6 shows the optical micrographs of selected regions along the flow path for the part obtained with different mold temperatures. The presence of colored bands can be ascribed to the high residual orientation frozen in some region of the part [24]. Close to the gate, the orientation is very high for all considered mold temperatures (Figure 6a,b). Orientation decreases with both distance from the gate and mold temperature. At the lower mold temperature, the cavity is not completely filled and the colored bands at the last flow length are less marked. At higher mold temperatures, the cavity is completely filled, and the lowest orientation can be found at the weld line. However, the colored bands are still present in the inner part of the curvatures, where a cold weld line forms.

**Figure 7:**
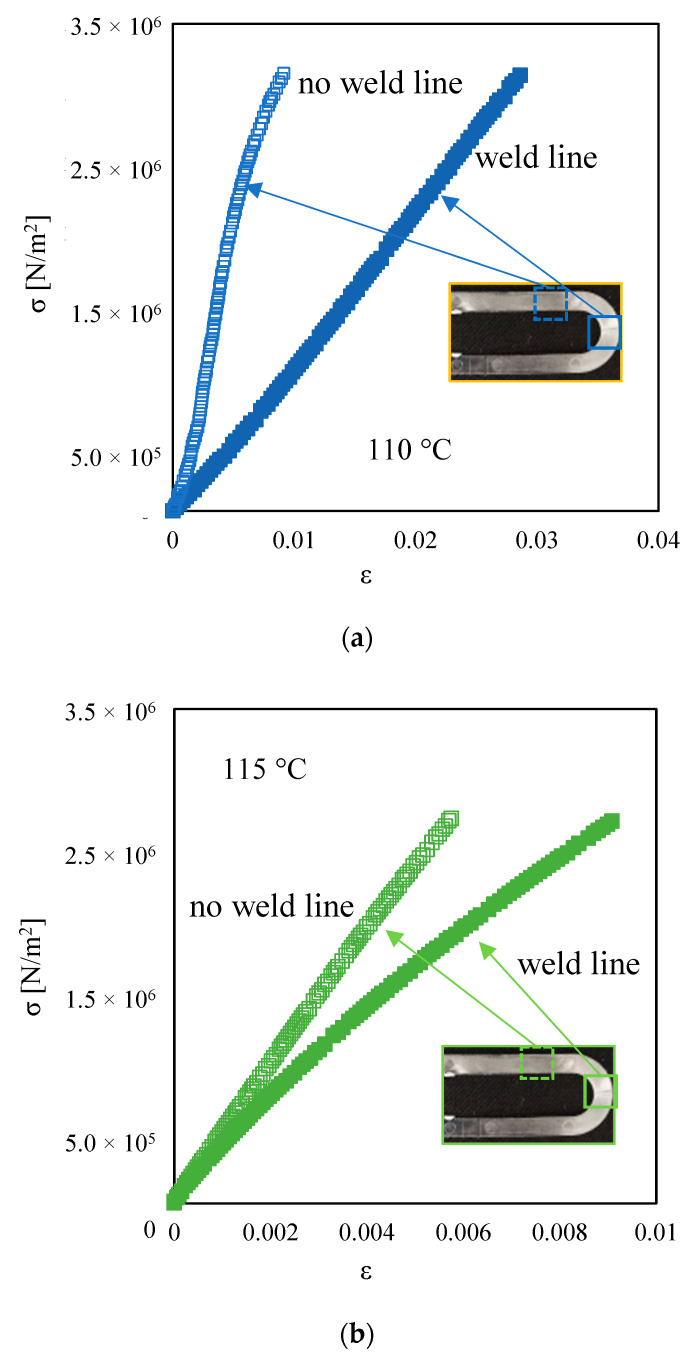
Comparison between stress–strain curves of samples obtained at (**a**) 100 °C and (**b**) 115 °C in the weld line area (filled squares) and in the other parts of the cavity without junction points (empty squares).

Figure 7 shows the results of the stress–strain analysis conducted on the parts obtained with 110 °C and 115 °C mold temperatures at two selected positions along the flow path. The stress–strain curve shows a higher slope for the specimen obtained before the weld line.

**Figure 8:**
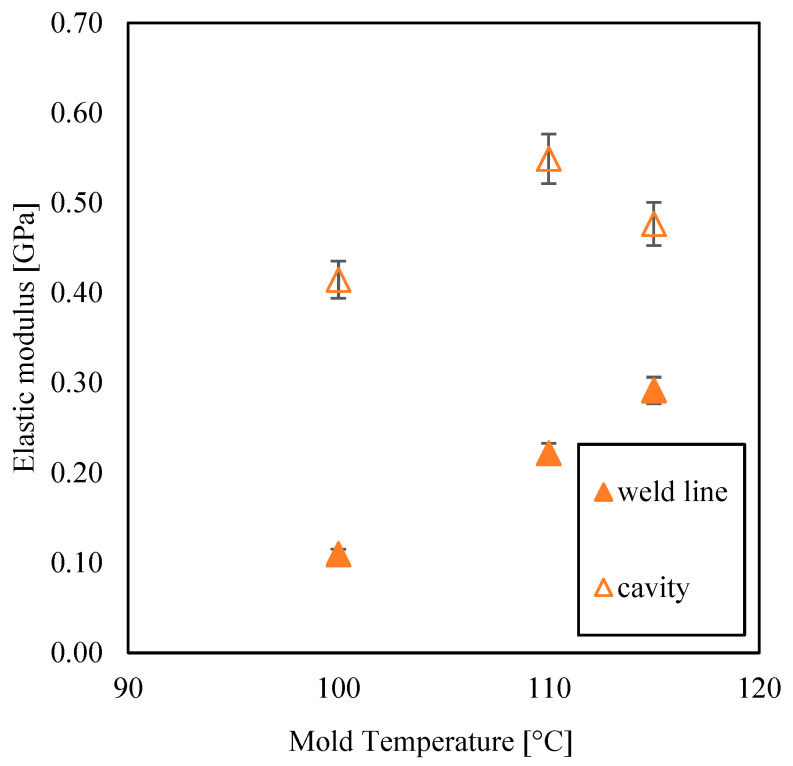
Elastic modulus in the weld line area (‘weld line’ series) and in the other parts of the cavity without junction points (‘cavity’ series) for samples processed with several mold temperatures.

Figure 8 shows the elastic modulus for the parts obtained at several mold temperatures, evaluated from the stress–strain curves in the two aforementioned positions along the flow path. The elastic modulus, E, was calculated from Equation (7) [4].

(7) $$E=\frac{∆\sigma }{∆\varepsilon }$$

The elastic modulus increases with the mold temperature in both selected positions; however, at the weld line, the elastic modulus is smaller than those observed in the region before the weld line. Tosello et al. [14] found a 20% difference between the elastic modulus measured in the continuous part and at the weld line. This work demonstrates that such a difference depends on the adopted mold temperature: the higher the mold temperature, the lower the difference is. As the mold temperature increases, the formation of a flowing weld line is favored; as a result, the elastic modulus at the weld line increases. In the region further from the weld line, the increase in the elastic modulus with mold temperature is due to a higher polymer structuring level [25]. 

## 4. µIM Simulations

MF simulations were conducted after selecting the piston position and travel speed according to the experimental data acquired during the µIM tests (see Table 2). The simulation does not account for material crystallization; however, the no-flow temperature (147 °C) in the rheological section compensates for the increase in viscosity due to the incoming crystallization. The no-flow temperature influences the slope of the pressure evolution at the injection point; thus, a calibration procedure was followed to assess this parameter. Such a procedure consists of determining the slope of the pressure curve during cavity filling and adjusting the no-flow temperature to obtain the correct slope and a filling length consistent with the experimental observations. 

**Figure 9:**
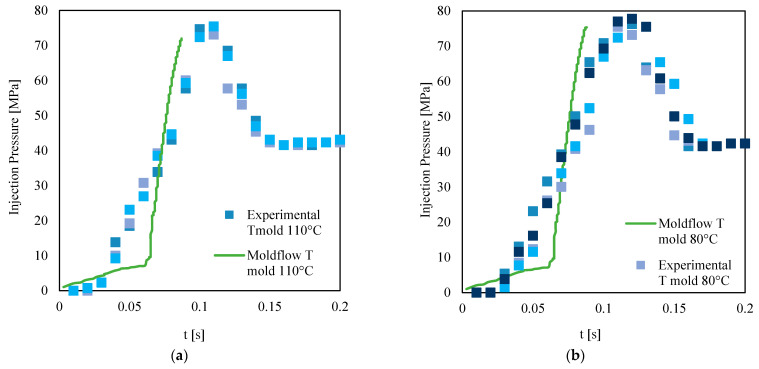
Pressure evolutions at the injection position obtained with 110 °C (**a**) and 80 °C (**b**) mold temperatures. Different colors are used to indicate test repetition.

Two mold temperatures were selected for such a calibration procedure; one is the minimum temperature at which the cavity appears completely filled, and the other one is a temperature 30 °C below the first one. Figure 9 shows the comparison between pressure evolutions obtained from the simulation and the experiments. The pressure evolutions obtained from the MF simulations are consistent with the experimental findings. 

It is possible to describe the filling through the pressure evolutions: the pressure shows low values up to 0.03 s, which corresponds to the time the melt needs to fill the sprue; the polymer fills the sprue and gate within 0.06 s; after that, the melt enters the cavity (a slope change is visible in the pressure curve). Once the melt enters the cavity, the pressure suddenly increases to the maximum value: 7 bar. At this point, the melt has filled the cavity as much as possible. Since packing is not set at the machine, the pressure decreases due to solidification. The residual pressure measured by the pressure transducer is related to the residual pressure of the melt present in the feeding chamber. The simulated pressure evolutions are consistent with the experimental ones; however, the pressure decrease after filling was not described (packing was not implemented in MF simulations).

**Figure 10:**
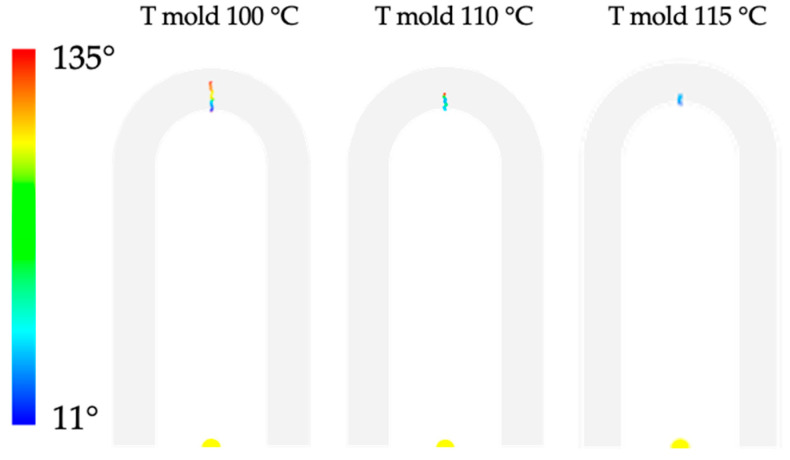
Angle between the flow front obtained by MF simulations conducted with several mold temperatures. For angles larger than 135°, MF does not show any results.

After calibration, several µIM simulations were carried out with the aim of analyzing the behavior of the polymer during filling and weld line formation. Figure 10 shows the angle at the melt front obtained from the MF simulations at different mold temperatures. Concerning weld line angle, when the angle between two flow fronts is below 135 °C, the line is called a cold weld line, or else it is a flowing weld line. 

**Figure 11:**
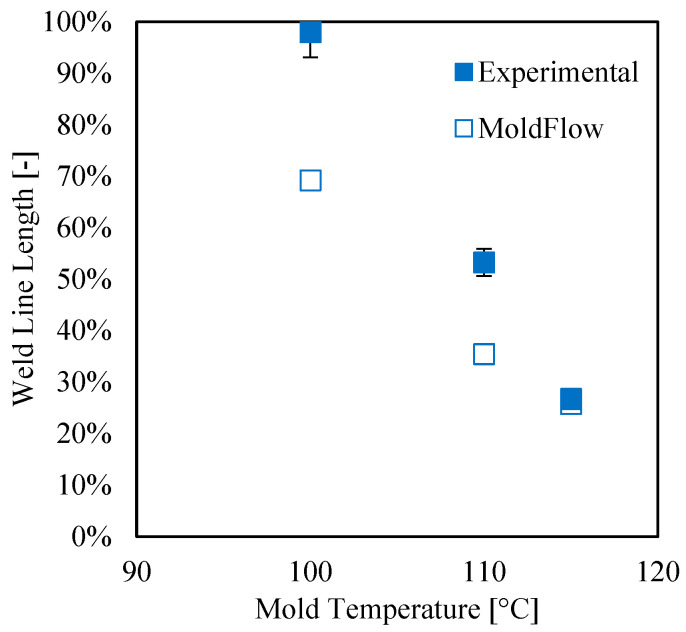
Length of the cold weld line for the tests conducted with different mold temperatures. Full symbols are used for the experimental points and empty symbols for simulations.

The predicted weld line location shows a good resemblance to the experimental location (see Figure 6). The simulations show that weld lines form at each considered mold temperature. Figure 10 shows that the length with an angle above 135 °C, namely the flowing weld line, increases with mold temperature; this finding is consistent with the experimental observations. Figure 11 shows the length of the cold weld line for the simulation tests conducted with different mold temperatures. 

The simulated cold weld line length is consistent with the experimental one when a 115 °C mold temperature is adopted; with lower mold temperatures, the simulated lengths are smaller than the experimental ones—in accordance with the literature on the conventional injection molding process [26,27]—probably because of the definition adopted by MF, which is obviously not applicable for experimental data. 

**Figure 12:**
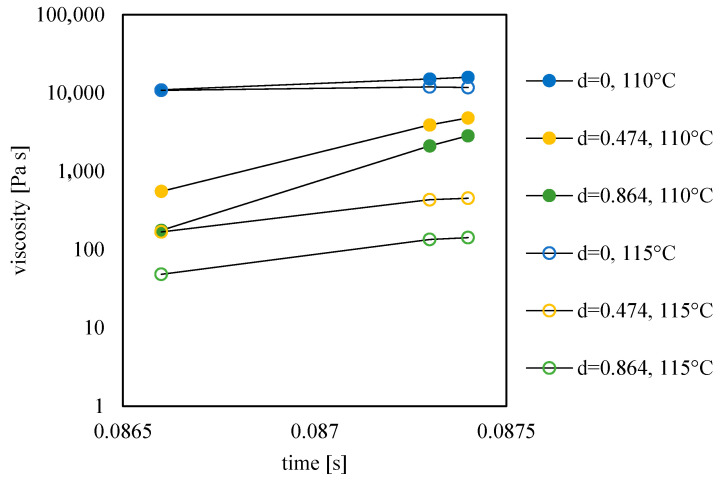
Viscosity evolutions at selected positions along the thickness (d is the normalized distance from the midplane) for the tests conducted with different mold temperatures.

The viscosity achieved by the melt during filling determines the interdiffusion at the flow front [28]. Figure 12 shows the viscosity evolutions obtained from MF simulations at a distance of 44.7 mm from the gate.

The melt front reaches the selected position at 0.0865 s from the injection time. As the process proceeds, viscosity increases due to the reduction of temperature and shear rate. At d = 1, which corresponds to the cavity surface, viscosity is the highest (10^7^ Pa s) since the no-flow temperature is suddenly reached in that position [21]. At further distances from the cavity surface, viscosity decreases due to the flow, according to the implemented Cross model [29]. Viscosity decreases with increasing mold temperature; such a decrease favors the formation of a flowing weld line (thus a reduction of the cold weld line length). This finding is consistent with those already reported in the literature on the effect of mold temperature on the mechanical performances at the weld line for conventional injection molding process [23].

**Figure 13:**
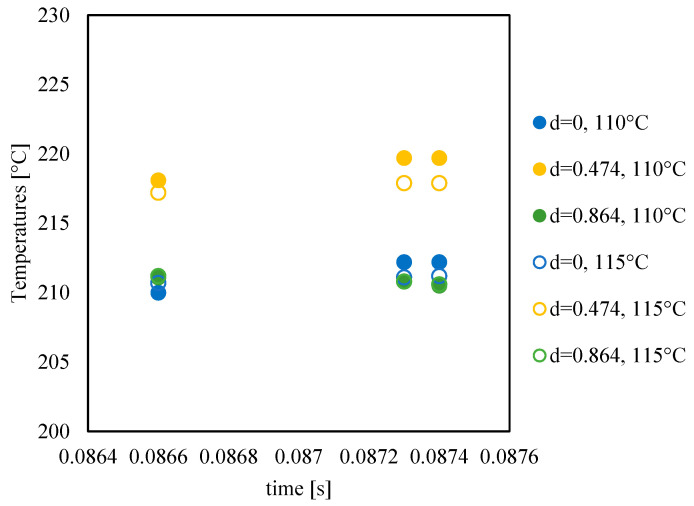
Temperature evolutions at selected positions along the thickness (d is the normalized distance from the core) for the tests conducted with different mold temperatures.

Figure 13 shows temperature evolutions, obtained from MF simulations, at selected positions along the thickness nearest to the weld line.

The position d = 1 is characterized by the temperature set for the mold, according to the high adopted heat transfer coefficient. In the other positions along the thickness, the temperature is above the melt injection temperature due to viscous dissipation; the temperature slightly increases with time at each considered position. The position at d = 0.474 shows the highest temperature according to both a reduction of the flow strength and the presence of viscous dissipation. It could be expected that the degree of orientation of polymeric chains and the thickness of oriented layers decrease considerably with mold temperature increases, which is induced by the stress relaxation of sheared chains and the reduced melt viscosity [29,30].

It has to be pointed out that the crystallinity evolution in flow conditions was not implemented in the material description adopted in MF simulations. Although the times of the process are very short, some flow-induced crystallization can take place before the complete filling of the cavity. The ongoing crystallization due to a strong flow may induce sudden solidification, hindering any flow at the weld line location. This approach leads to some advantages since the characterization of the crystallization kinetics is challenging, especially for fast-crystallizing polymers (in the presence of nucleation agents); furthermore, it allows for safe calculation times (calculation time could be very long, more than twice, when crystallization is accounted for). The appropriate selection of the no-flow temperature allows us to obtain reliable results in terms of both pressure evolutions and weld line location and quality, even in the absence of flow-induced crystallization.

## 5. Conclusions

An extensive exploration was conducted into the impact of mold temperature on weld line characteristics and strength, employing a combination of experimental and simulation methodologies. The investigation illuminated several critical insights:

(i) The mold temperature was found to be a pivotal factor influencing the efficacy of cavity filling by the polymer material. The experimental results revealed that achieving complete cavity filling necessitated a mold temperature of at least 100 °C, while lower temperatures often led to premature solidification and incomplete filling; (ii) mold temperature emerged as a determinant in weld line formation. The experimental observations, coupled with simulation outcomes, highlighted the development of distinct weld line types, specifically cold and flowing weld lines. The influence of mold temperature was particularly pronounced, with higher temperatures fostering the creation of flowing weld lines; (iii) the interplay between polymer orientation, mechanical properties and mold temperature was another significant facet. The rise in mold temperature corresponded to an augmentation in the elastic modulus, especially at the weld line. This phenomenon was attributed to the formation of flowing weld lines with superior mechanical attributes.

The simulation approach, relying on Moldflow, allowed for a better understanding of the condition, and position, at which the weld line took place. In particular, the simulations showed that the angle between the flow front is lowest in the inner part of the curvature, thus inducing cold weld lines, in line with the experimental observation. 

The crystallization aspect within the simulation model could potentially bridge the remaining gap between predicted and actual outcomes, leading to more accurate predictions and informed process improvements in micro-injection molding.

An understanding of mold temperature’s effects on the mechanical performances at the weld line is crucial at the industrial level since it allows the correct design of the mold and, in particular, of the injection points whose position determines the weld line location. 

## Figures and Tables

**Table 1:** Predicted flow lengths at several mold temperatures evaluated from Equations (3)–(6).

$T_{w}$ [°C]	$L_{f}$ [mm]	Filled Volume [%]	$t_{f}$ [s]
70	35.0	85.0	0.11
80	37.7	88.8	0.13
90	40.7	93.0	0.15
100	44.2	97.9	0.18
110	48.6	104.1	0.23

**Table 2:** Experimental data generated during µIM tests.

Time [s]	Piston Position [mm]	Travel Speed [mm/s]	Flow Rate [cm^3^/s]
0.01	17.1	10	2.5
0.02	7.0	10	2.5
0.03	6.7	30	7.6
0.04	5.9	80	20.4
0.05	4.6	100	25.4
0.06	3.2	130	33.1
0.07	2.2	100	25.4
0.08	1.5	70	17.8
0.09	1.3	40	10.2
0.10	1.0	0	0

## Data Availability

The data are unavailable.
